# Methodology of a Study Assessing a New Curriculum Designed to Teach Australian Final Year Medical Students How to Assess Patients’ Spiritual Needs

**DOI:** 10.1007/s10943-025-02545-7

**Published:** 2026-01-10

**Authors:** John Wenham, Megan Best, David W Kissane AC

**Affiliations:** 1 School of Medicine, University of Notre Dame Australia, 160 Oxford St Darlinghurst, Sydney, NSW Australia; 2 Institute for Ethics and Society, The University of Notre Dame Australia, 104 Broadway, PO Box 944, Sydney NSW, Australia; 3 Chair of Palliative Medicine Research, The University of Notre Dame Australia, Sydney, NSW Australia

**Keywords:** Spiritual history taking, Medical education, Communication skills training, Spiritual care, Curriculum

## Abstract

Many physicians believe that spiritual care is a necessary component of holistic medical care. Despite the apparent importance of spiritual care, very few Australian medical schools include such content in their curricula. We used data from two previous research projects to develop the evidence base for an Australian medical school curriculum for spiritual care training. This curriculum paper describes the content and delivery of pilot workshops, assessment of their efficacy, along with details of the learning journey for both the students and the medical educator. Learning needs were identified in a pre-workshop interview with a simulated patient. Each student had the opportunity to demonstrate their new spiritual history-taking skills, during the workshop and in a formative assessment in the weeks following their training. Our aim for this paper is to share our methodology and our curriculum. We also describe how we approached the assessment of its efficacy, the outcome of which will be presented in a subsequent paper. We hope this will assist other educators in adopting this model of teaching spiritually focused holistic patient care.

## Introduction

Spiritual care is a vital component of holistic medical care (Rombola, 2019). Svalastog and colleagues defined holistic health as, “a relative state in which one is able to function well physically, mentally, socially, and spiritually to express the full range of one’s unique potentialities within the environment in which one lives” (Svalastog et al., 2017). Many physicians today support this view of spiritual care as a necessary component of holistic medical care (Puchalski et al., 2009). However, in the seventeenth-century Enlightenment, significant moves away from practising medicine in such a paradigm were made; instead, it was deemed that the church alone should look after the spiritual. Locke argued that, “the care of Souls cannot belong to the Civil Magistrate, because his Power consists only in outward force; but true and saving Religion consists in the inward persuasion of the Mind, without which nothing can be acceptable to God” (Locke, 2013). The medical profession focused on the physical component of patient care, seeing the body as a machine; when it malfunctioned, it needed repair. By the mid-nineteenth century, medicine was infused by a physicalist approach (Kaplan, 2005). This included the need to demonstrate the efficacy of physical interventions on changing outcomes; a scientific evidence-base is more difficult to construct for the psychosocial and spiritual domains (Gonçalves et al., 2017).

It is not surprising that in the early twenty-first century, with their secular and humanistic approach to medicine, very few Australian medical schools include spirituality in their curricula. A survey of 147 GPs practising in Australia regarding spiritual history-taking skills in holistic consultations identified a number of barriers. These included insufficient time, personal discomfort with the subject matter, and concerns from doctors about crossing boundaries into spiritual areas that are not perceived to be appropriate for their profession to address. Most participants recognised a lack of knowledge and skills and thus desired further training, especially in spiritual history-taking. There was strong support for this being included in Australian medical education (Rombola, 2019). We set out to address this gap in the medical school curriculum by designing and delivering a workshop to equip medical students, and therefore future doctors, to include spiritual assessment in the holistic care of their patients.

We used data from two previous research projects to develop the evidence base for an Australian medical school curriculum for spiritual care training: (1) a review of the international literature exploring examples of curricula seeking to equip medical students to navigate this important component of holistic care (Wenham et al.,^2021^); and (2) a survey of final year Australian medical students’ experiences of spiritual care and spiritual history-taking during their journey through medical school, where we sought feedback on the findings from our initial review (Wenham et al., 2024). Data extracted from both projects were used to inform the construction of a three-hour workshop on spiritual care for final year medical students.

This paper describes the content and delivery of the pilot training workshops, the methodology used to create them, an overview of the assessment design and details of the learning journey for both the students and the medical educator. In a subsequent paper, we will present the quantitative evaluation which demonstrated the educational benefit of this curriculum (Unpublished reference 1); in a final qualitative paper the success of our teaching pedagogy will be presented (Unpublished reference 2).

## Methodology

### Design

Each spiritual care workshop was preceded by a single 12-min Observed Structured Clinical Examination (OSCE) station (Harden, 2016; Ledford et al., 2014) which incorporated a significant spiritual component. This had the dual purpose of assessing each student’s baseline communication skills prior to the teaching episode, as well as demonstrating their learning needs in the spiritual care domain. During the workshop, the students were given the opportunity to reflect on their performance; the tutor then provided constructive feedback on the tasks that had been done well and identified areas for improvement.

Research data building the evidence base for patients’ holistic care needs and doctors’ learning needs were used to bolster the argument for developing spiritual care skills. Our pedagogy employed reflective inquiry and lecture content, group discussions and clinical case examples. Role play was incorporated to promote mastery of spiritual history-taking. This skill development was confirmed by a second OSCE assessment, which took place after the workshop to consolidate learning, and allowed the tutor to give students feedback to drive continual improvement.

### Participants

Final year medical students were invited to participate in a study assessing the impact of the spiritual care workshop on student communication skills which was conducted entirely in a rural setting. The offer to join the study was made via e-mail and subsequent phone call by the educational support officer at the local clinical medical school. Participant information sheets were provided to interested students who were invited to a meeting with the primary investigator where they were offered further explanation of the study and an opportunity to ask questions. The students were advised of the potential benefits of learning new skills from the workshop and also gaining feedback on their communication skills during the pre- and post-workshop interviews with a simulated patient before asking for written informed consent from those who agreed to participate.

We reasoned that, as modern medical students need encouragement to attend tutorials and clinical placements, they would need to be informed of the positive impact on patient care of engaging with a voluntary research project, especially the chance to practise OSCEs used to examine them at the end of the year. Providing opportunity for the investigator to present the nature and purpose of the research to potential participants was beneficial in gaining their consent. Endorsement of the value of the research from senior colleagues was also essential.

Workshops commenced in April 2024 in the outback town of Broken Hill at the University of Sydney’s Department of Rural Health (UDRH), followed by a further workshop at the University of Notre Dame’s clinical school in Ballarat. Ethics approval for this study was obtained from the Human Research Ethics Committee at The University of Notre Dame Australia (Ref. 2023-099S).

### Objectives of the workshop

The spiritual care workshop had seven clear objectives:

1. Use the pre-workshop role play to demonstrate to the students their skills gap in spiritual care
2. Present key evidence for and cultural relevance of spiritual care
3. Understand the nature and content of holistic care
4. Evaluate the interaction between patient beliefs and health care choices
5. Analyse the key beliefs of the main world religions
6. Demonstrate how to take a spiritual history from a colleague and receive feedback.
7. Use post-workshop role plays to ground the assessment in the experiential interaction with simulated patients.

### Workshop Overview

The formative OSCE prior to the workshop, which was recorded for later independent standardised assessment, provided the opportunity for student reflection and tutor feedback, resulting in the identification of learning needs. Then, there was presentation of evidenced-based literature in the domain of spiritual care. Spirituality was defined and discussed. Two models of spiritual history taking were presented: the Belief/Practice/Community model, abbreviated from the SPIRITual history (Maugans, 1996), and the HOPE model (Anandarajah & Hight, 2001); comparisons were made and discussed. The concept of learning a variety of questions to keep in the student’s "communication skills toolbox” for use when required was explained. Opportunity to practise the skill of spiritual history-taking with a colleague, and to receive feedback, was given. A link was made between culture, religion, and health care choices. Four major religious world views were presented and explored. Vignettes of relevant case studies were presented and discussed in small groups. All students returned in the weeks following to consolidate their learning in a second formative OSCE, which was again assessed independently for the skills present. Further feedback on the communication skills demonstrated was given in person by the investigator to each student once they had completed the task.

### Workshop Pedagogy and Content

We wanted to develop a clear link between spiritual wellbeing and their core curriculum. We wanted them to be inquisitive and to have their preconceptions challenged. Alternative medical models were summarised briefly, from the simple biomedical, to the more inclusive bio-psycho-social (Engel, 1977) and then person-centred care (McCance & McCormack, 2017). Humans are the integration of body, mind and spirit with physical, psychological and spiritual dimensions (Karff, 2009). It was underlined throughout the workshop that it is unlikely that students will need to take a spiritual history from every patient, as it may not be relevant in every context. Parallels were made with the place of a sexual history, which is elicited only when clinically relevant. However, students were assured that having the skills for such interventions in their communication skills toolbox will make them a more effective holistic medical practitioner in the future.

### Presentation

The following material was presented in the first half of the workshop: definitions of spirituality, data on world religions and evidence-based literature on the benefits of spiritual care. The content and student responses are detailed below.

#### Definitions

Two definitions of spirituality were presented for student reflection and group discussion:

1. “Spirituality is the aspect of humanity that refers to the way individuals seek and express meaning and purpose and the way they experience their connectedness to the moment, to self, to others, to nature, and to the significant or sacred” (Puchalski et al., 2009). This was the product of a United States (US) consensus conference.
2. “Spirituality is a dynamic and intrinsic aspect of humanity through which we make sense of reality and find meaning. A person's spirituality orientates them in space and time. Spirituality is expressed through beliefs, values, traditions and practices.”(Balboni et al., 2022) This definition is used by chaplains at Westmead Hospital, Sydney, to inform conversations with patients. We have also used it in a first-year medical school tutorial at the University of Sydney.

#### Group Discussion

Discussion of definitions of spirituality generated varied responses, creating the moment in the workshop where the topic seemed to gain traction as students realised we were not simply talking about religiosity.

**Fig. 1:**
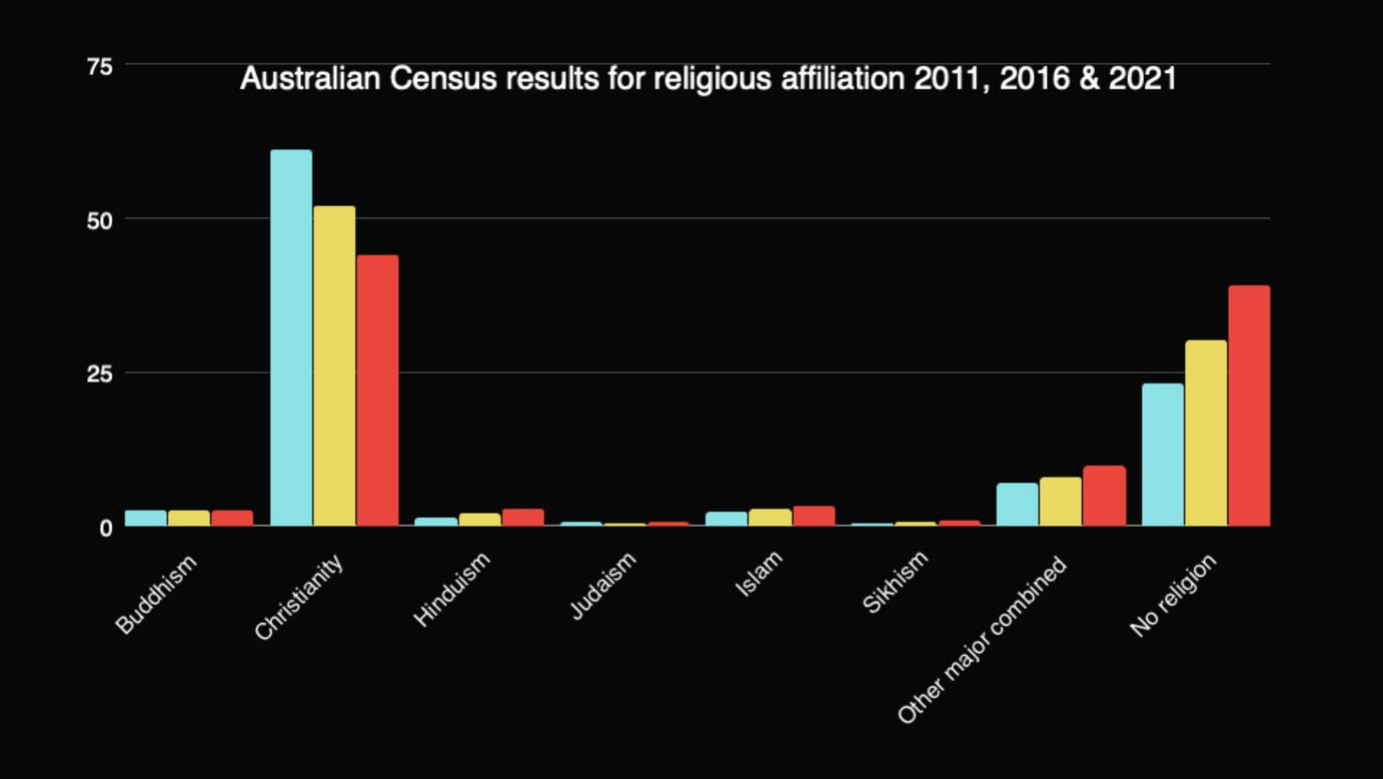
World religion in Australia

Data from the 2021 Australian census on Religious Affiliation were shared and compared with 2016 and 2011 findings (Australian Bureau of Statistics 2021). In 2021, 44% Australians identified as Christian; Islam was the second most prominent religion with 813,000 adherents (3%) and Sikhism was the fastest growing religion with a 200% increase in 10 years (Fig. 1). The commonly forgotten fact that Australia’s Constitution (1900) was founded on Christian principles and ethics was also presented.

#### Evidence for Inclusion of the Spiritual Domain in Patient Care

This section of the workshop could have been quite didactic. By testing students’ ability to make an educated guess on what some of these statistics might be, and encouraging reflection on the research findings, we enabled a greater level of engagement and participation.

The following information was presented and discussed:

A clear separation exists between doctors’ and patient’s perceptions in the domain of spiritual care needs (Best et al., 2015); according to this study, 70% patients are interested in discussing spirituality with their physician whilst only 15% doctors over a wide range of settings include it. There is clear evidence that spiritual well-being improves health outcomes (Burlacu et al., 2019; Park et al., 2015) but barriers to these discussions in standard consultations include insufficient knowledge and training (Best et al., 2016). Evidence suggests spiritual care should be included in the core teaching of our medical schools (Puchalski et al., 2014; Schonfeld et al., 2016).

Students and facilitators discussed this strong endorsement of the holistic care model, “It is critical that we as physicians and health care providers listen to all aspects of our patients’ lives that can affect their decision making and their coping skills” (Puchalski, 2001). The discussion of intimate topics requires the “creation of a safe space” (Best et al., 2016) and advanced communication skills, for example, the ability to understand and respond sensitively to the patient’s distress, whether it is of a medical, emotional, or spiritual nature (McEvoy et al., 2014). The barriers to doctors taking a spiritual history include time, personal discomfort and boundary concerns (Rombola, 2019).

Further encouragement to engage with this workshop was offered: Clinicians could, “profitably pay more attention to spirituality” (Williams & Sternthal, 2007). Patients report that good communication is important at all levels of doctor–patient interaction; this involves assessing unmet needs, gaining permission, being culturally sensitive, seeking understanding, responding empathically, and summarising what has been learnt. This will improve patient satisfaction and reduces patient anxiety (Kissane et al., 2012). Finally, there is good evidence from the USA of how to enable undergraduate medical students to address patients’ spiritual needs (King et al., 2004).

#### 2024 Final Year Medical Student Survey Findings

We then presented the findings from the author’s previous study examining the views of Australian final year medical students on spiritual care training (Wenham et al., 2024). This paper found that, from a sample of 260 students, only 11% had observed a physician take a spiritual history from a patient; 10% of the students had been given the opportunity to take a spiritual history themselves; and 68.5% of the cohort had never had any training in spiritual history taking or spiritual care. Only 11.2% were against discussing spiritual matters. Barriers to engaging with such matters included insufficient knowledge and training, lack of time, and fear of crossing into inappropriate areas within the doctor/patient relationship. Enablers included it being a patient priority, either culturally or situationally important, and having had spiritual care training. Almost 56% of the survey sample affirmed that medical students should receive training in this field as part of their core curriculum, with a small majority preferring delivery in later clinical years.

#### Holistic Medicine

The transition from the theoretical to the practical part of the workshop was managed by asking the group to discuss the components of complete wellbeing. Students were then challenged to consider what questions they might ask when taking a spiritual history. Their experiences in the first OSCE station were used to discuss what challenges they had identified. Many found it confronting when they realised they didn’t have the skills in their communication toolbox to navigate the scenario particularly well. For many students, this became a driver for learning.

#### Presentation of Belief/Practice/Community Framework for Spiritual History Taking

The substantive part of the training workshop commenced with a presentation of a simple model to elicit a spiritual history: belief, practice and community (see Box 1) (Maugans, 1996) and then the HOPE model (see Box 2) (Anandarajah & Hight, 2001). Subsequently, the students were asked to consider how a Buddhist or Hindu person might respond to these questions.

**Box 1:**
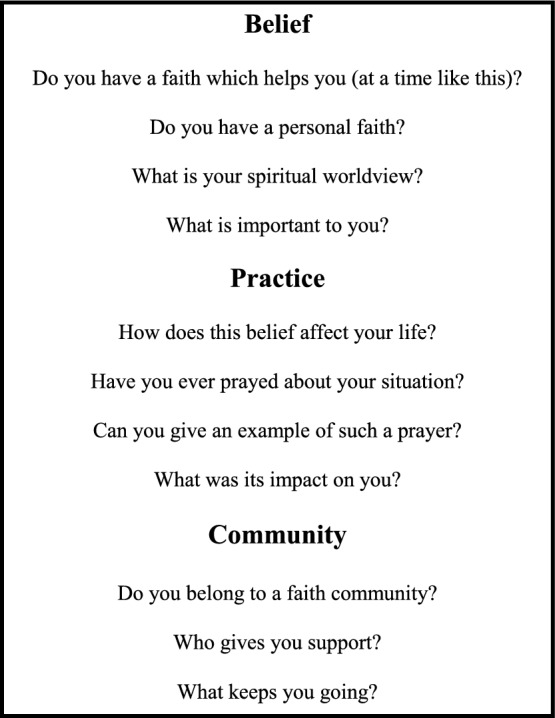
The Maugan’s model for spiritual assessment

**Box 2:**
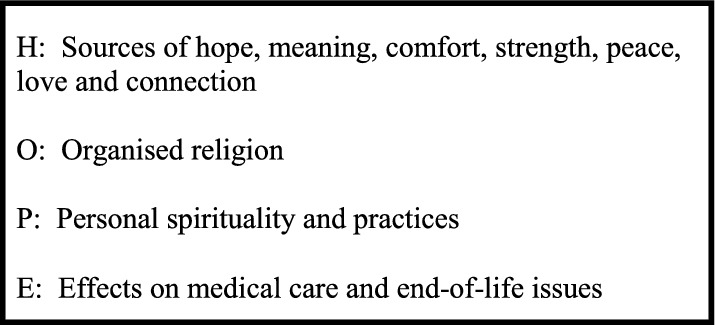
HOPE model of spiritual assessment

#### Spiritual History Taking Practice and Feedback

The next step was for the students to form pairs and take a spiritual history from each other. At this point they were given a two-sided *aide-mémoire,* which they could use and annotate as they trialed the questions with one of their peers. The relevant merits of both sets of questions and the effectiveness of responses were then discussed. The concept of seeing the spiritual history questions as a toolbox to dip into rather than a list to work through was explained. The students were encouraged to consider selecting questions from the menu that they might employ at the second OSCE (OSCE 2), when they were likely to need to take a spiritual history. In the interval between the workshop and OSCE 2, they were encouraged to practise using, and test out the relative effectiveness of, these new questions.

### Religious Beliefs and Healthcare Choices

The group was asked to consider the following questions:

1. Should we consider religious beliefs and healthcare choices?
2. What relevance do patient beliefs have in healthcare?
3. What are the benefits of accommodating these?
4. What are the potential pitfalls?

The significance of religious beliefs, both in the patient’s culture and in the world view they might bring to ethical decision making and healthcare choices, was discussed. Summary slides for each of the four major (in terms of number of adherents) world religions were discussed: Buddhism, Christianity, Hinduism, and Islam.

### Vignettes for History Taking and Response

In groups of three, the students were each given a vignette to role play: one patient, one doctor, and one observer to give feedback. Five minutes was allowed for each role with instructions to feedback and then rotate. Broader group feedback was then facilitated for each case. The vignettes are given below:

### Case 1

A 30-year-old married couple come to see you after two years of trying to conceive. They have had no previous pregnancies and want to discuss their options going forward. Taking into account their Christian worldview, how will you help them navigate the options available to them?

#### Case 2

A 45-year-old Muslim Type 1 diabetic gentleman comes to see you prior to Ramadan. He is under pressure to conform to the daily fast from sunrise to sunset for the next 30 days. He is concerned about the possibility of hypoglycaemia.

#### Case 3

A 50-year-old lady comes to see you and breaks down in tears. Her 25-year-old daughter has just had a termination of pregnancy. She has found herself overcome with guilt as she had the same procedure herself 20 years ago. Explore her physical, emotional and spiritual needs.

#### Workshop Conclusion

There are various clinical situations where it will be relevant to take a spiritual history from a patient. Triggers that should alert the clinician to do so include end of life scenarios, sharing difficult diagnoses with patients, or a consultation involving anxiety about, or expressions of, guilt. Each participant was asked to share one key learning point from the workshop; these were wide and varied as repetition was not permitted to prevent them all having the same idea. Instructions were given on how to complete the post-workshop questionnaire. Arrangements for the subsequent OSCE station were then explained. Following OSCE 2 feedback was given to them as a part of the learning process.

#### Evaluation: Fidelity of OSCE Assessment

Simulated patients rehearsed the role play scenarios with the facilitator until uniformity of presentation was achieved. Two assessors, independent of the workshop leader, evaluated the recordings of the participants’ pre- and post-workshop OSCE using a standardised checklist developed with a pilot candidate and the simulated patients. Inter-rater reliability was set at > 60%. Participant feedback sheets and satisfaction ratings were summarised. These outcomes were shared individually with participants at a later date. Ordinal data from each workshop were assessed for effect sizes to demonstrate uniformity across workshops and overall efficacy of the curriculum.

## Discussion

### Group Dynamics

Four distinct groups were recruited to the pilot study of a spiritual care workshop i. It is important to create a curious group dynamic in order to facilitate learner inquiry (Hopfenbeck et al., 2022). The students that had clearly self-identified spirituality or religiosity, or who had a genuine interest in understanding the spiritual needs of their patients, exhibited a higher personal investment in the workshop from the start. Some grew in inquisitiveness once the ‘spirituality = religion’ myth had been debunked.

Focus was maintained throughout the group workshops by a number of methods. The facilitator sought to elicit opinions from all members, including quieter students, in order to develop a mutually supportive dynamic. Small groups of two or three were formed to discuss the deeper questions and encourage participation as they grappled with a new topic. The promise of an agreed time for a break, with refreshments provided, prevented the waning attention that is normal in a three-hour training session.

### Facilitator Skills

The facilitator of these pilot workshops had 20 years’ experience of teaching medical students and junior doctors in one-to-one and small group settings. He had delivered shorter tutorials on holistic care and spiritual history taking in inter-professional workshops as part of an Enhancing Rural Inter-professional Cultural Health (ENRICH) programme; this programme looks at holistic multi-disciplinary patient care and enables health students to appraise the value of a coordinated approach (Bolte et al., 2012). In 2017, four sessions expanding on the cultural relevance of the four main world religions were added to the ENRICH programme and were well received.

It is anticipated that in a future rollout of the curriculum into medical schools in Australia, New Zealand and possibly the UK, facilitator training may need to occur in order for a reasonable replication of this material along with an engaging delivery. The workshop itself presented an additional curriculum to the students, beyond that on which they would be examined in their summative examinations. Therefore, it was necessary to be mindful of the voluntary nature of their participation and acknowledge this before and after they consented and during their attendance.

A variety of methods were used to encourage students to understand the workshop’s ability to enhance their overall learning and communication skills. Firstly, whether they had signed up out of a sense of duty to join their colleagues, possibly through fear of missing out, or through inquisitiveness; in many cases the pre-workshop OSCE exposed their deficiencies in spiritual history taking. This was a key driver of their learning. Secondly, a clear explanation of the essence of holistic patient care was given. Thirdly, the evidence from literature on the subject persuaded the more sceptical students of its relevance given that patients express a strong desire for it to be part of their medical care. Fourthly, time spent defining spirituality brought clarity to those who might shun a programme that simply focused on religion and health.

Good facilitation skills in small group learning are essential so that the process is learner-centred; didactic delivery might be teacher-centred but it rarely leads to the instillation of passion toward the subject matter in the learners because their role is simply too passive. In this workshop, learners were encouraged to interject and to ask questions as the presentation progressed; clear times for small group discussion were identified in the slides that accompanied the material being shared. The two trigger words “role play” have the potential to induce panic and push-back in students. Our preferred approach is to invite them to practise the material together in twos and threes whilst avoiding the trigger words. Communication skills teaching lends itself to an interactive approach. Students and facilitators alike recognise the importance of turning passive absorption of theory into active application of skills in short sequence whilst the new knowledge is fresh.

Expertise is important in a facilitator; however, the best approach to oral delivery is as a guide who reveals the material to the students in a manner that builds on previous understanding. This process is known as scaffolding (Zackariasson, 2020). If student attention begins to wane, it is an opportunity for the tutor to consider whether the material is either too easy or too challenging for them. There is also a need to consider the alternative possibility that they might be hungry or tired and how that can be mitigated.

The post-workshop OSCE allowed the students to test out their new knowledge of spiritual histories and their communication skills by eliciting one. This is an effective way of consolidating learning. At the same time, it gave the researchers the opportunity to appraise the impact of the pilot workshop. There was a need to adapt the patient script when a student came forward prior to the second OSCE to advise she thought she would find the content emotionally challenging as her father had recently had a stroke. This was a potential critical incident that required some minor adaptation of the patient script to preserve equanimity.

### Group Evolution

Four groups of final year medical students took part in the spiritual care workshop. Over the course of three hours, most of the participants were observed to have an increased interest in and understanding of the relevance of spirituality within the practice of holistic medicine. The reframing of student perspectives was enabled through a number of methods which included placing OSCE 1 prior to the workshop; this allowed for self-identification of knowledge and skills gaps. Sharing evidence-based research findings that show spiritual care is important to patients and has a beneficial impact on health added to the argument that it should be a learning need that they should consider and subsequently want to address.

Whilst the three-hour workshop was a relatively short period in which to teach the students how to approach a spiritual history, they were given a summary framework (belief/practice/community) and an in-depth assessment tool (HOPE); either of these could be applied in isolation or in a hybrid fashion depending on the circumstances of the patient, and the preferences of the student. During the workshop, they had the opportunity to test these questions out on each other immediately after they had been presented, and later in the workshop using some short vignettes. This allowed the students to demonstrate creativity as they navigated an unfamiliar domain.

The final consolidation of learning occurred within 4 weeks of the workshop when they were informally assessed in the second OSCE. It is true that the workshop could be run in isolation without the OSCE stations. However, there is clear evidence that formative OSCEs not only consolidate student learning, but allow the instructor to evaluate their performance and provide feedback (Ledford et al., 2014). Thus, the OSCE becomes an effective part of a reflection-based pedagogy.

### Limitations

The setting for this pilot study was in rural Australia which limited the number of final year medical students on long-term placements suitable for the longitudinal nature of this study. The study itself requires up to five hours of student time. It is possible that the sample may therefore be skewed towards students with a genuine interest in the subject matter. Involvement in research proved to be quite a commitment for the students who agreed to participate, despite the competing pressures of study and clinical placements on their time. However, this was mitigated by the offer to learn a new subject and have the opportunity to practise their OSCE skills and receive feedback.

## Conclusion

The spiritual care workshop that has been presented in this paper is recommended by the authors for adoption in medical school curricula. The time commitment will vary from half to one day per student depending on whether the OSCE stations are incorporated on the day of the workshop or not. Evaluation findings and evidence for the efficacy of the workshop as a learning programme will be reported in subsequent papers. Introduction of this programme marks significant progress in rebalancing the scales away from the focus on physical and psychological patient management that has beleaguered medical programmes since the Enlightenment.
